# Modification of the postural pattern of the trunk by means of a set of shoe lifts

**DOI:** 10.1186/1748-7161-9-S1-O3

**Published:** 2014-12-04

**Authors:** Michele Romano, Kjell Roger Heitmann, Alessandra Negrini, Matteo Mastrantonio, Fabio Zaina, Stefano Negrini

**Affiliations:** 1 ISICO - Italian Scientific Spine Institute, Milan, Italy; 2 DIERS International GmbH, Schlangenbad, Germany; 3 University of Brescia - IRCCS Don Gnocchi, Brescia, Italy

## Background

After the medical evaluation of a spinal disease, the indication of a shoe lift (SL) is prescribed in case of a recognized improvement of some specific outcome.

## Aim

The purpose of this study is to measure sagittal and coronal imbalance of the trunk and modification of the hump magnitude, in standing position, in response to the use of a SL (for this study a series of SLs).

## Design

Observational study.

## Methods

We evaluated 35 consecutive female patients visited in ISICO Institute for spine diseases (scoliosis or hyperkyphosis). With the patient in a standing position, we carried out a set of tests performed with a three-dimensional rastereography (DIERS Formetric) with different SLs (5mm, 10mm, 15mm.) placed alternatively under both feet. We assessed the variations of these different postural outcomes: modification of the hump, sagittal and coronal imbalance of the trunk.

## Results

The statistical analysis of the seven acquisitions shows that:

- In the coronal plane, the average variation of the inclination of the line between C7 and the center of the sacrum is 1.8mm ± 0.32.

- In the sagittal plane the average variation of the inclination of the line between C7 and the center of the sacrum is 1.2 ± 0.44.

- In the horizontal plane the average variation of the main hump is statistically significantly different (reduction of average 3.1° ± 1.7) when the SL is placed under the opposite foot (left hump – right foot).

## Conclusion

A previous study focused on the observation of postural changes of the trunk in response to the use of a series of SLs demonstrated that the trunk does not change the specific postural characteristics.

This study that completes that previous evaluation, shows that the use of SLs alternatively placed under both feet does not show a statistical significative difference of the typical postural pattern of the trunk in coronal and sagittal plane. In the horizontal plane the use of a SL can reduce the hump magnitude.
